# Use of Intense Pulsed Light to Mitigate Meibomian Gland Dysfunction for Dry Eye Disease

**DOI:** 10.7150/ijms.44288

**Published:** 2020-06-01

**Authors:** Abhishek Suwal, Ji-long Hao, Dan-dan Zhou, Xiu-fen Liu, Raja Suwal, Cheng-wei Lu

**Affiliations:** 1 Department of Ophthalmology, The First Hospital of Jilin University, No. 71 of Xinmin St., Changchun, Jilin Province, 130021, China.; 2 Department of Radiology, The First Hospital of Jilin University, No. 71 of Xinmin St., Changchun, Jilin Province, 130021, China.; 3 Department of Radiology, Koshi Zonal Hospital, Biratnagar, Province No. 1, Nepal.

**Keywords:** dry eye disease, intense pulsed light, meibomian gland dysfunction, mechanism of action, meibum

## Abstract

Dry Eye Disease (DED) is a common ocular condition that needs prompt diagnosis and careful treatment interventions. If left untreated, it can lead to numerous sight-threatening complications, including ulceration of the cornea, blepharitis, alterations of the tear film, conjunctivitis, and in severe cases, may lead to scarring, thinning, and even perforation of the cornea. Intense pulsed light (IPL) is a non-laser high-intensity light source that has shown to play a valuable role in dry eye disease. Recent evidence from various research works has shown that IPL modifies the mechanism of meibomian gland dysfunction (MGD), which helps to relieve the symptoms of DED. In this review, we demonstrated the mechanism of action of IPL, including its benefits on DED. The emerging evidence shows that the role of IPL in DED is novel and therapeutic. These results direct us to conclude that IPL is a potentially beneficial tool and essential future therapy for dry eye disease. Advances in the treatment of DED will lead to a better quality of life. However, tools to recognize potentially severe side effects of DED earlier in order to treat or prevent them must be developed.

## Introduction

An intense pulsed light (IPL) device is a non-laser high-intensity light source that uses a high-performance flash lamp to produce a non-coherent light output of large wavelength, usually, in the range of 500 to 1200 nm. Light pulses generated by most modern devices are produced by bursts of electrical current passing through a xenon gas-filled chamber 1. IPL use in the medical field relies on the basis that specific targets for energy absorption (chromophores) are capable of absorbing energy from this broad spectrum of light wavelength (absorptive band) without exclusively being targeted by their maximum absorption peak. The working principle of the IPL is based on selective photo thermolysis, in which thermally mediated radiation damage is limited to have chosen epidermal and dermal pigmented targets at the cellular or tissue structural levels 2. The initial published report of the use of an IPL device in ophthalmology dates back to 2002 when Toyos et al. observed improvements in MGD related Dry Eye Disease (DED) patients 3,4.

DED is a common ocular condition that needs prompt diagnosis and careful treatment interventions. If left untreated, it can lead to numerous sight-threatening complications, including ulceration of the cornea, blepharitis, alterations of the tear film, conjunctivitis, and in severe cases, significant drying of the eye may lead to scarring, thinning, and even perforation of the cornea 5. Henceforth, early diagnosis and proper treatment are necessary to prevent further complications and to restore the vision as well as maintaining the integrity of eyelids. The cause of dry eye varies from obstructive such as age-related disorder to evaporative such as meibomian gland dysfunction (MGD) 6.

IPL uses electromagnetic waves of desired wavelengths to dilate the capillaries, causing them to involute 7. This results in the suppression of the leaked inflammatory mediators, which interrupts the vicious cycle of inflammation and improving symptoms of dry eye. It also works with the help of thermal pulsation for many patients 8. In the incidence of chronic inflammation, the meibum's composition changes to include more monounsaturated fats. Those fats have a significantly higher melting point of close to 45 ºC - warmer than body temperature 9. This meibum does not melt into the tear film's lipid layer as it should, and it clogs the glands. Thermal pulsation therapy combines sustained heat and pressure to liquefy the meibum and clear the glands. Manually expressing glands is less effective, uncomfortable for patients, and potentially scarring 10. Thermal pulsation is gentle and effective. With these mechanisms, we have come to an understanding that IPL helps to relieve the symptoms of dry eye 11. This literature review aims to compare a good deal of research work done in the field of IPL and their associated results in treating DED (**Table 1**).

## Mechanisms of action

### Thrombosis of abnormal blood vessels

The energy produced by IPL, directed towards the eyelid, is absorbed by hemoglobin and is transformed into heat and causes the localized destruction of superficial blood vessels 12. Obliteration of atypical erythematous blood vessels reduces a significant reservoir of inflammatory mediators, thus removing a significant source of inflammation from the eyelids and meibomian glands 13.

### Eradication of Demodex and reducing the bacterial load

Demodex is an ectoparasite that usually burrows deep into sebaceous and meibomian glands to feed on the meibum secretions 14-16. A direct consequence of the spread of Demodex is the dramatic increase in bacterial load on the eyelids 17, particularly *Bacillus oleronius* 18. The pigmented exoskeleton of Demodex comprises a chromophore that absorbs IPL energy 19. Histology analysis confirmed that IPL treatment induces coagulation and necrosis of Demodex 20. By eradication of Demodex, IPL could help decrease the microbial load on eyelids and possibly break the malicious cycle of inflammation. Thus, the annihilation of Demodex and reducing the bacterial load on the eyelids 19-21 is the most significant approach towards reducing the incidence of MGD in DED patients 22.

### Photobiomodulation

**Figure 1:**
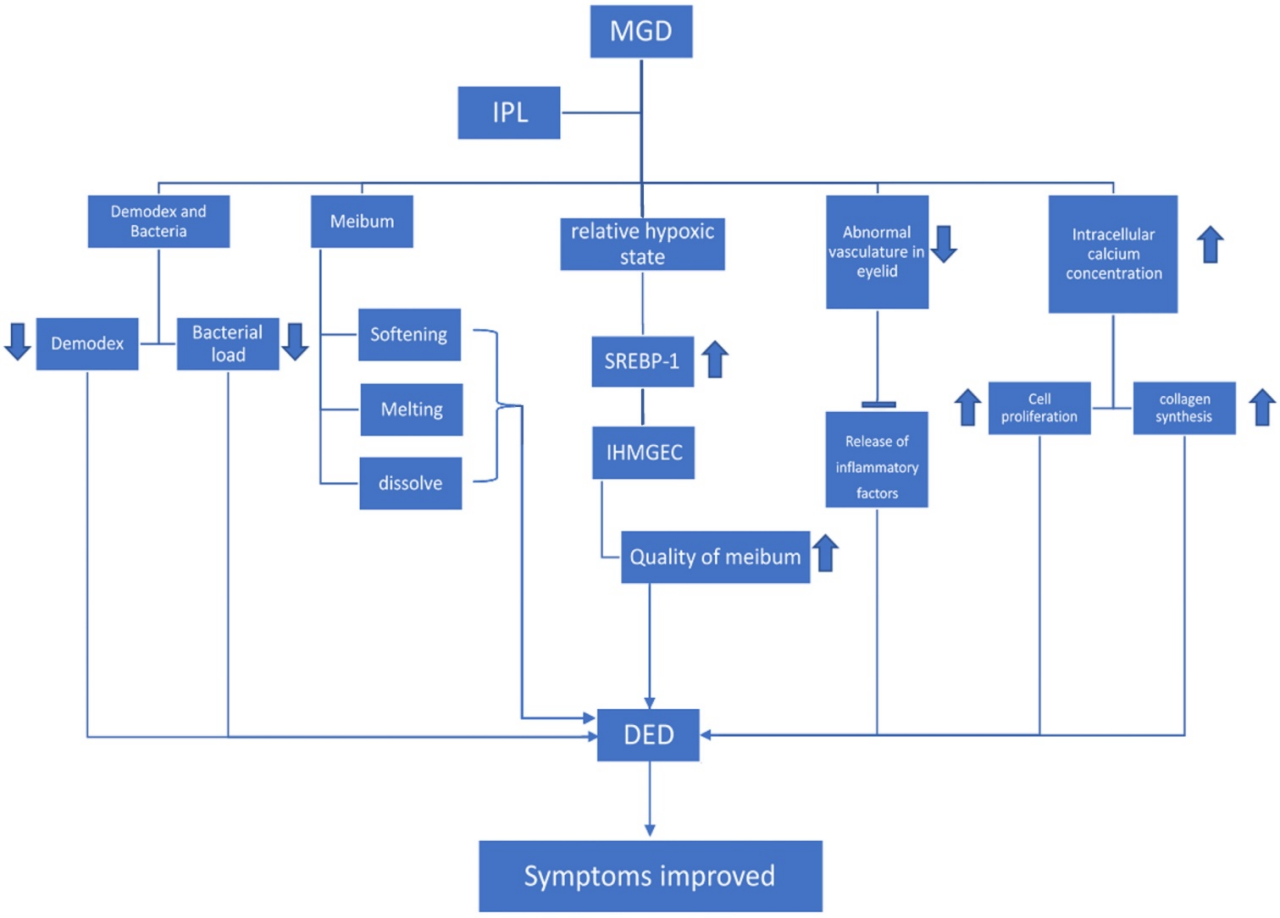
MGD, when treated with IPL, helps reduce the Demodex as well as bacterial load. Whereas in the meibomian gland, IPL helps to soften, melt, and dissolve the meibum. IPL helps create a relative hypoxic state in the MG, which stimulates the expression of SREBP-1 in differentiating IHMGECs, thereby improving the quality of lipid content in meibum. IPL also helps to reduce the abnormal vasculature in the eyelid, thus inhibiting the release of inflammatory factors. Intracellular calcium concentration is increased, which leads to an increase in cell proliferation and collagen synthesis. These mechanisms help in relieving the symptoms of DED.

Photobiomodulation is a procedure by which light in the visible and infrared portions of the electromagnetic spectrum induces intracellular changes at the gene and protein levels 23. IPL produces a photochemical cascade, inducing changes in the redox properties of components along the mitochondrial respiratory chain, leading to faster electron transfer and, hence, to an increase in adenosine triphosphate production 24. In the case of fibroblasts, cell proliferation is boosted, and collagen synthesis is increased 25; skin-homing T cells are recruited 26; local blood flow is increased; macrophages cells are activated 27; epidermal keratinocytes increase the secretion of pro-inflammatory or anti-inflammatory cytokines and chemokines (**Figure 1**). The ability of IPL to activate fibroblasts and enhance collagen synthesis is the basis for the efficacy of skin rejuvenation as well as MGD related DED treatment 28,29. At the eyelid skin level, this effect could contrast the natural tendency of the skin to lose rigidity and elasticity with aging. This process could lead to poor apposition of the lid margins and incomplete blinks, resulting in reduced meibum secretion and increased tear evaporation.

### Effects on meibum

The temperature of the eyelid significantly regulates the flow of meibum secretion. IPL induces heating of the meibomian glands and dissolving the meibum, activation of fibroblasts, and enhancing the synthesis of new collagen fibers, thrombosis of damaged blood vessels 12 below the skin surrounding the eyes. Bäumler et al. suggested that in medium and large blood vessels (>150 μm), a single IPL pulse of 30 ms duration raises the temperature at the center of the vessel to 80 °C-90 °C, above the temperature required to cause coagulation and thrombosis. In contrast, in small (60 μm) blood vessels, the temperature may reach only 45 °C-70 °C, depending on the flow 30. This rise in temperature is insufficient to destroy blood vessels. However, it is probably enough to raise the temperature of eyelid skin (and meibomian glands) by a few degrees, perhaps above the phase-transition temperature. Even if brief, this thermal response could be enough to unclog the meibomian glands and reinstate their ability to excrete meibum during blinking 31. According to the different IPL patterns, "Optimal Pulse Technology" (OPT) was more effective in improving meibomian glands function in lower eyelids and partial tear film signs than "Intense Regulated Pulsed Light" (IRPL) IPL treatment 32.

### Effects on pro- and anti-inflammatory molecules and suppression of MMPs

Factors that adversely affect tear film stability and osmolarity can induce ocular damage and initiate an inflammatory cascade that generates a robust immunological response, which in turn, may cause further damage at the ocular surface, creating a self-perpetuating inflammatory cycle 33. IPL has the potential to hinder the inflammatory cycle, by up-regulation of anti-inflammatory cytokines, or down-regulation of proinflammatory cytokines, or both. The inflammatory cascade in dry eye is extremely complex and incompletely understood. However, it is plausible that at least part of the beneficial effect of IPL on DED patients occurs by interfering with the positive feedback loop underlying the inflammatory cycle of this pathology 31.

Intrusion with the inflammatory cycle by regulation of anti-inflammatory agents and MMPs, diminishing the turnover of skin epithelial cells and reducing the risk of physical obstruction of the meibomian glands, and changes in the levels of Reactive oxidative species helps in preventing the symptoms of dry eye 34.

While any one of these mechanisms of action has likely to explain the effect of IPL on DED, it is also possible that multiple mechanisms of action play various roles. As IPL becomes more frequently used in the treatment of DED, the specific involvement of each of these modes of work will be further elucidated.

### IPL induces hypoxic condition on meibomian gland

Loss of relative hypoxic state of the meibomian gland plays a significant role in the pathogenesis of MGD. Acinar atrophy in a human MGD is linked with a thickening of the basement membrane. This anatomical development may be used to describe a compensatory response to decrease O2 delivery from adjacent vessels and to restore the relative hypoxia needed for optimal MG function 35. The latest research by Yiu et al. has demonstrated that human meibomian glands exist in a relatively hypoxic condition, and any alteration in this state dramatically affects the condition of the meibomian gland 36. As we can assume that heat emitted by IPL creates a hypoxic surrounding in the meibomian gland, IPL may be very beneficial for treating DED through this mechanism. Hypoxia does not influence immortalized human MG epithelial cells (IHMGEC) numbers in basal or proliferating culture conditions but does stimulate the expression of SREBP-1 in differentiating IHMGECs. Hypoxia also significantly increases DNase II activity and IHMGEC terminal differentiation. IPL is known to reduce telangiectases significantly 11, and the closure of these excessive vessels may help the MG to restore its hypoxic environment and normal function 37. Thus, hypoxia may also play a role in the reported effectiveness of Intense Pulsed Light (IPL) for MGD treatment.

### Effects on mucin and corneal nerve

Mucins are large molecular glycoproteins formed by abundant sugar chains linked to a core protein called apomucin, with 50% to 80% of their mass comprised of carbohydrates 38. Mucin has several essential roles on the ocular surface such as maintenance of lacrimal fluid, lubrication of the ocular surface to ease smooth blinking, formation of a smooth spherical surface for good vision, facility of a barrier for the ocular surface, and trapping and removing pathogens and debris 39,40. The tear film has two distinct layers inside which the aqueous layer contains secreted mucin MUC5AC dispersed within it 40. Xue et al. found that there were no changes in the MU5AC expression using Conjunctival impression cytology after IPL treatments 41. Study done in the nerve fibre density and dendritic cell density of the corneal sub-basal layer also showed no change achieved by IPL treatment 41. Even though the changes were not statistically significant, we think that further studies with more extended treatment periods and bigger data are needed.

## IPL associated benefits

### Intense pulsed light in patients with refractory meibomian gland dysfunction

More and more evidence suggested that intense pulsed light when used to treat MGD patients helps to relieve the symptoms of dry eye disease 11,42-46,47,48-55. Arita et al.'s study was to assess the safety and efficacy of intense pulsed light (IPL) joint with meibomian gland expression (MGX) for the treatment of refractory meibomian gland dysfunction (MGD). Her result suggested that the combination of IPL and MGX enhanced homeostasis of the tear film and ameliorated ocular symptoms in patients with a refractory MGD and is thus a promising modality for the treatment of this condition 43,44. IPL treatment improved meibomian gland function, stabilized the tear film, and decreased ocular surface inflammation. Meibum quality, lid margin abnormality, meibum expressibility, tear film break-up time (TBUT), ocular surface staining, and the Ocular Surface Disease Index (OSDI) drastically improved whereas poor meibum expressibility and short TBUT were associated with more significant improvement in the OSDI after IPL 56,57.

IPL treatment, along with meibomian gland probing, showed positive results in treating patients with refractory obstructive meibomian gland dysfunction 58,59. Studies done by Huang et al. on refractory obstructive MGD found out that compared with IPL or MGP alone, the combination MGP-IPL produced the best results in relieving all signs and symptoms and helping patients attain long-lasting symptom relief 60.

### Risk vs. benefit associated with IPL treatment

A study done by Gupta et al. demonstrated that IPL therapy for evaporative DED is a safe procedure. The positive change in objective clinical examination findings and subjective OSDI scoring data suggest that IPL is a safe and effective treatment for patients with evaporative DED. There was a significant increase in oil flow score and TBUT. No significant variations in intraocular pressure or acuity were noted. There were no cases of adverse ocular effects 61. No adverse effect after the IPL treatment, along with significant improvement in the symptoms of MGD, was noted in some studies 62. IPL treatment is further confirmed to be safe and effective in Chinese MGD patients with darker skin types (Fitzpatrick skin types III-IV) 63.

Another study done by Rong et al. demonstrated that intense pulsed light applied directly on eyelids combined with meibomian gland expression helps in treating MGD. The study was to determine the efficacy and safety of intense pulsed light (IPL) that was applied directly to the eyelids, and MGX helped to treat MGD. There were no cases of adverse ocular effects. This result clearly shows that IPL combined with MGX safely and effectively treated MGD 59.

Nevertheless, we have to keep in mind that the beam of light produced can be focused on the specific area which selectively damages specific targets in the area being treated 64 (e.g., capillaries, brown spots or tattoo pigment in the skin) allowing them to be removed altogether or the area to be replaced by new cells—depending on the desired treatment. Effects produced by IPL may sometimes be undesired and cause risks such as burns, blistering, and pain 65. Severe symptoms may include keloids and skin pigmentation. Thus, the physician should inform the concerned patients for the risk vs. beneficial effects of IPL therapy before moving forward with the treatment.

### Effect of intense pulsed light therapy on tear proteins, lipids, and inflammatory marker in meibomian gland dysfunction

IPL helps to improve the symptoms of DED by regulating the concentrations of total lipids, triglycerides, cholesterol, and phospholipids in the tear. Ahmed et al. noted that significant improvements were observed in tear protein concentrations and molecular weight after IPL therapy. The most pronounced effect was in the molecular weight of tear lysozyme, lactoferrin, and albumin. On thin‑layer chromatography, the tears in patients with MGD had significantly lower amounts of anionic phosphatidylethanolamine, phosphatidylinositol, and phosphatidylserine, but amounted zwitterionic neutral phospholipid phosphatidylcholine were normal. These anionic phospholipids showed remarkable recovery after IPL therapy 66. IPL improved tear protein and lipid content and composition. Various studies have shown a promising effect of decreasing interleukin-6, interleukin-17A, and prostaglandin E2 in tear fluid of DED patients after IPL treatment 43,56,67. In addition, they suggested that the decrease in these inflammatory factors was linked to the beneficial improvement of clinical symptoms and signs 67,68. These reductions on the inflammatory factors were associated with changes in corneal staining scores, which is indicative of improvements in ocular surface epithelial damage 69. Some studies have shown that changes in IL-6, IL-17A, and IL-1β levels were lowest at about one week after IPL, which was sooner than the appearance of clinical outcome peaks at one month 70. This suggests that changes in tear cytokine levels may be more sensitive indexes than clinical signs to show the effects of IPL (**Table 2**).

### Effect on the meibum of MGD by IPL

Numerous studies have shown that IPL helps loosen the clogged meibum by thermal pulsation therapy. Godin et al., in his research work, suggested that MGD is a vital contributor to dry eye disease in patients with Sjogren disease and should not be overlooked when considering treatment options. Thermal pulsation helped the meibum to release its clogged ducts. A thermal pulsation is a therapeutic option for patients with Sjogren disease who have MGD and dry eye symptoms 71, which can directly improve the quality of meibum. Another study by yin et al. showed that OSDI, TBUT, meibum quality, MG expressibility, and MG dropout improved after treatment. The MG microstructure indexes, including meibum, the MG acinar longest diameter (ALD), MG acinar unit density (AUD), and the positive rate of inflammatory cells (ICs) around glandular structures, were significantly improved by IPL treatment. These findings suggest that IPL treatment improves the symptom of MGD in DED patients. It also improves the associated ocular-surface indexes, MG function, and MG macrostructure as well as eyelid hygiene.

Furthermore, IPL treatment mainly improves MG microstructure and decreases MG inflammation in MGD patients 58,72. Studies conducted by Chhadva et al. on the meibum of the MGD patients found out that MGD leads to a change in meibum quality and quantity that leads to evaporative dry eye and ocular surface disruption, causing dry eye symptoms in some individuals 73. These changes can be controlled by the use of IPL in a systematic way so that the difficulty faced by the patient can be minimized 68.

### Significant improvement in the first IPL treatment

Evaluation of the short-term effect on the first IPL treatment combined with meibomian gland expression showed significant improvement in the symptoms of meibomian gland dysfunction 74. Considerable improvements were observed in single visits after the initial IPL treatment. Compared to baseline, the signs of the eyelid margin, meibomian gland secretion quality, and expressibility were significantly improved at every visit after treatments. There were no local or systemic adverse effects observed in any patient after IPL treatment 75. Patients exhibited increased oil flow and tear break-up time with an associated decrease in corneal and conjunctival staining 36,71. A study done by Gao et al. showed that inflammatory markers such as IL-6 were lower at one week of the treatment than that of one month, showing that the IPL treatment starts showing its effect from the earliest 70.

## Conclusion and Future Aspects

DED is a crucial issue in ophthalmic medicine. It has increased in occurrence with the increasing development of screen-based gazettes such as computer and mobile because of which, the blinking rate of fellow human being decreases. Several other factors, such as dust and pollution, also play a significant role in the epidemiology of DED. This literature review aimed to evaluate the use of IPL in MGD and its associated benefits in DED. The reviewed literature suggests that there is an advantage to the use of IPL on ophthalmic cases such as DED. Reduction in symptoms of DED, as well as MGD in conjunction with an improvement in living conditions has been widely documented throughout the literature. These all lead to the conclusion that the use of IPL shall provide vital effectiveness in the field of DED. These findings highlight the potential of IPL as a novel therapeutic procedure in DED. With the evolving trends in medical science, IPL, with current therapeutic management, could be used as a vital tool for MGD as well as DED. Its role as the therapeutic machine could be explored for the welfare of human beings. Current research supports the use of IPL, as discussed above; however, a continuation of current research with consistent and strengthened methodologies will help to justify its use and application in clinical practice.

## Figures and Tables

**Table 1:** Summary of studies on IPL for DED Therapy

Year	Design	Patients (n)	IPL sessions (n)	Dry eye symptom	BUT	Meibomian function	Reference
2020	Prospective randomised double-masked placebo-controlled	58	5	Improved OSDI, SPEED, SANDE	Improved	Improved	41
2020	Prospective randomized double-masked sham-controlled	114	3	Improved OSDI	Improved	Improved	62
2020	Prospective controlled randomized	29	3	Improved OSDI	Improved	Improved	32
2020	Retrospective	43	Mild group: 2; Moderate group: 3;Severe group: 4	Improved OSDI in mild and moderate atrophy patients, not severe ones	Improved in mild and moderate atrophy patients, not in severe ones	Improved in mild and moderate atrophy patients, not in severe ones	45
2019	Prospective controlled randomized	45	3	Improved SPEED	Improved	Improved	60
2019	Prospective controlled randomized	45	8	Improved SPEED	Improved	Improved	43
2019	Prospective controlled	12	1	Improved OSDI	Improved	-	66
2019	Prospective comparative	40	3	Improved OSDI	Improved	-	63
2019	Prospective noncomparative	30	3	Improved OSDI	Improved	Improved	56
2019	Prospective noncomparative	19	3	Subjective symptoms improved	Improved	No statistically significant changed	48
2019	Prospective noncomparative	30	3	Improved OSDI	Improved	Improved	76
2019	Case-control	82	1	Improved OSDI	Improved	Improved	70
2019	Retrospective	28	3	Subjective symptoms improved	Improved	Improved	49
2019	Retrospective	25	-	Improved OSDI	Improved	Improved	22
2018	Prospective, randomized, double-masked, controlled	44	3	Improved SPEED	Improved	Improved	59
2018	Prospective controlled randomized	28	3	Improved SPEED	Improved	Improved	57
2018	Prospective noncomparative	31	4-8	Improved SPEED	Improved	Improved	44
2018	Prospective noncomparative	26	3	Subjective symptoms improved	Improved	-	68
2018	Prospective noncomparative non-randomized	17	4	Improved OSDI	Improved	Improved	69
2018	Prospective Cohort	35	3	Improved OSDI	Improved	Improved	72
2017	Prospective, randomized, double-masked, controlled	44	3	Improved OSDI, SPEED	Improved	Improved	67
2017	Prospective, randomized, double-masked, controlled	44	3	Improved SPEED with no statistical difference	Improved	Improved	74
2017	Prospective interventional noncomparative	40	4	Improved SPEED	Improved	Improved	50
2017	Prospective noncomparative	36	4	-	Improved	-	47
2016	Prospective, open label	40	4	Subjective symptoms improved	Improved	Improved	75
2016	Retrospective	100	4	Improved OSDI	Improved	Improved	61
2015	Prospective controlled	28	3	Improved OSDI, SPEED	Improved	-	37
2015	Retrospective noncomparative case series	91	7	Subjective symptoms improved	Improved	-	3

**Table 2:** Summary of Reviewed Articles on the tear film and inflammatory markers

Study	Design	Patients (n)	IPL sessions (n)	Inflammatory marker (down-regulated)	Tear protein and lipid	Tear film
Ahmed et al.,^66^ 2019 J Ophthalmic Vis Res	Prospective controlled	12	1	Prevent inflammatory mediator secretion	Improved	Lipid concentration of tear film improved
Arita et al.,^43^ 2019 Ocul Surf	Prospective controlled randomized	45	8	IL-6, IL-17A, prostaglandin E2	Improved	Improved homeostasis of the tear film
Choi et al.,^56^ 2019 Sci Rep	Prospective noncomparative	30	3	IL-4, IL-6, IL-10, IL-17A, and TNF-α	Improved	Tear film break-up significantly improved after treatment.
Liu et al.,^67^ 2017 Am J Ophthalmol	Prospective, randomized, double-masked, controlled	44	3	IL-6, IL-17A, prostaglandin E2	The correlation between the expression of IL-17A, IL-6 in protein levels showed no statistically significant	Improved tear film quality
Karaka et al.,^68^ 2018 Eur J Ophthalmol	Prospective noncomparative	26	3	IL-6, IL-17A, prostaglandin E2	Improved	Significant improvement in tear film quality
Seo et al.,^69^ 2018 Cont Lens Anterior Eye	Prospective noncomparative non-randomized	17	4	Down-regulated	Improved	Instability resolved
Gao et al.,^70^ 2019 Int J Ophthalmol	Case-control	82	1	IL-6, IL-17A, and IL-1β	Improved	Improve tear film (TBUT)

## Abbreviations

IPL: Intense Pulsed Light
MGD: Meibomian Gland Dysfunction
DED: Dry Dye Disease
MGX: Meibomian Gland Expression
CFS: Conjunctival Fluorescein Staining
TBUT: Tear film Break-up Time
OSDI: Ocular Surface Disease Index
IHMGEC: immortalized human MG epithelial cells
SREBP-1: sterol regulatory element-binding protein 1
